# Supplementary material for: Gene drive mosquitoes can aid malaria elimination by retarding Plasmodium sporogonic development
Source: Sci Adv. 2022 Sep 21;8(38):eabo1733. doi: 10.1126/sciadv.abo1733 (PMC9491717; doi:10.1126/sciadv.abo1733)
Supplement: Supplementary file 1 — Figs. S1 to S7 Tables S1 and S2 [file sciadv.abo1733_sm.pdf]

Science Advances
AAAS

Supplementary Materials for

Gene drive mosquitoes can aid malaria elimination by retarding *Plasmodium* sporogonic development

Astrid Hoermann *et al.*

Corresponding author: George K. Christophides, g.christophides@imperial.ac.uk;
Nikolai Windbichler, n.windbichler@imperial.ac.uk

Sci. Adv. **8**, eabo1733 (2022)
DOI: 10.1126/sciadv.abo1733

The PDF file includes:

Figs. S1 to S7
Tables S1 and S2
Legends for supplementary files S1 and S2

Other Supplementary Material for this manuscript includes the following:

Supplementary files S1 and S2

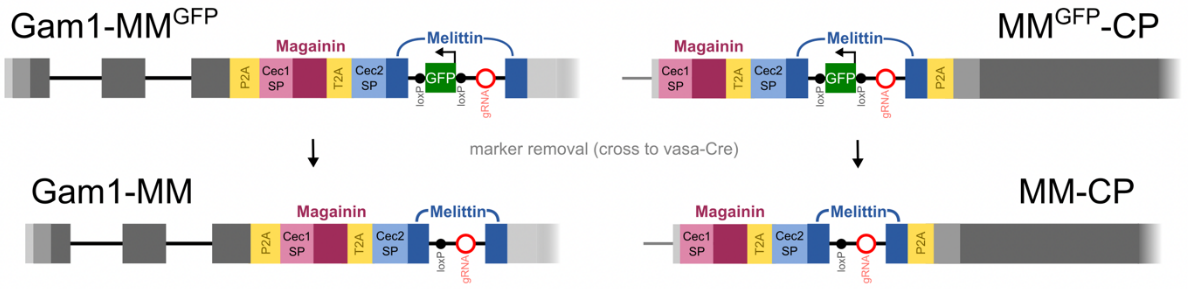

Fig. S1.
Schematic showing the inserted transgene constructs at the Gam1 or CP loci and the predicted exon structures following the excision of the GFP marker gene by Cre recombinase. The eGFP marker driven by the 3xP3 promoter and trypsin 3' UTR is shown in a simplified way and not to scale.

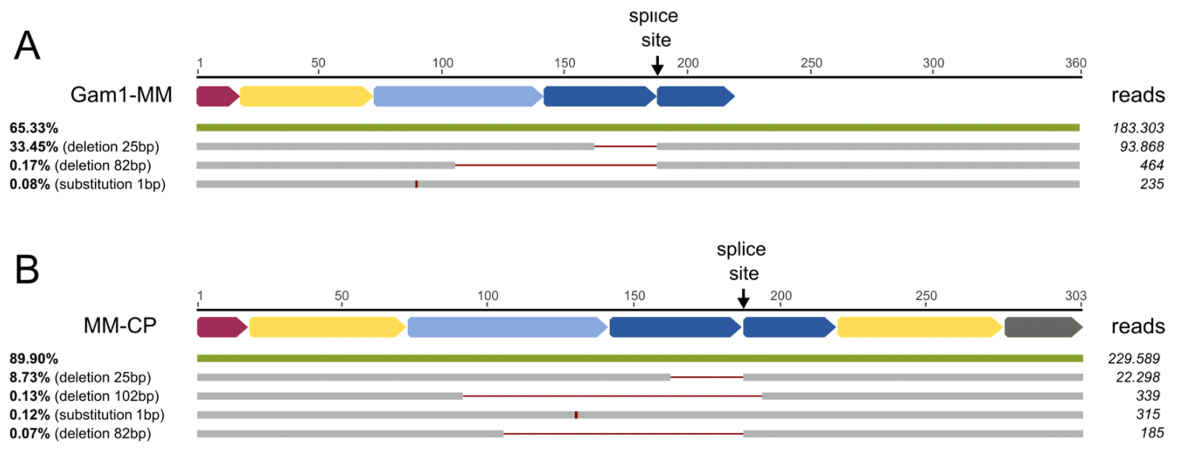

Fig. S2.

Alignment of sequenced cDNA amplicons to reference sequences representing the expected splicing outcomes for Gam1-MM and MM-CP. The splice site is indicated by the black arrow, the relative distribution of predicted variants (grey) representing at least 0.07% of all reads is shown on the left and the corresponding number of reads on the right.

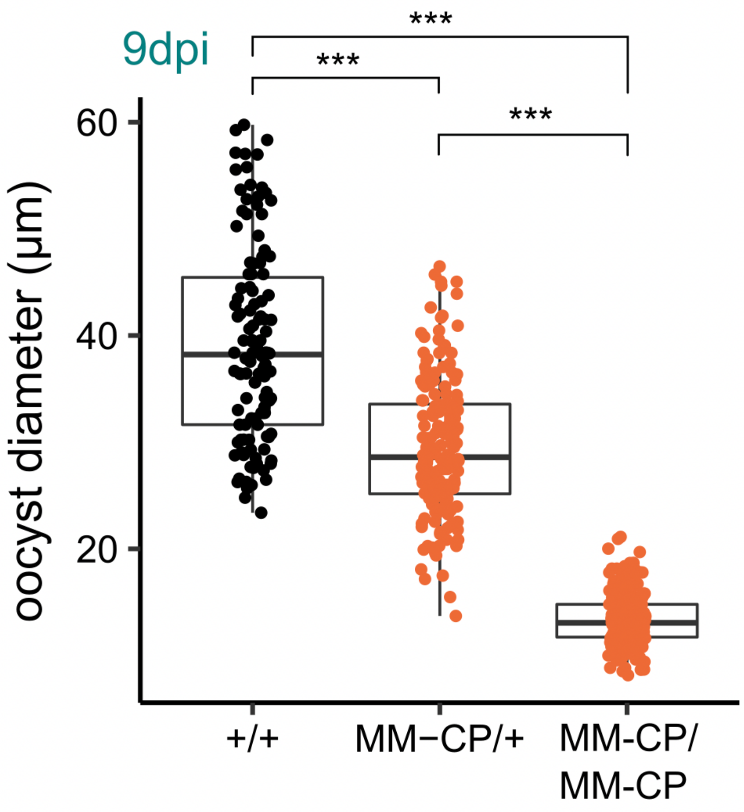

Fig. S3.

Quantification of *P. falciparum* oocyst diameter in F2 individuals following a backcross of strain MM-CP to the Ifakara strain and an F1 sibling intercross from 3 pooled biological replicates. Non-transgenic (+/+), hemizygous (MM-CP/+) and homozygous (MM-CP/MM-CP) individuals were identified by individual PCR genotyping after oocyst size had been determined. Statistical analysis was performed by a t-test assuming unequal variance.

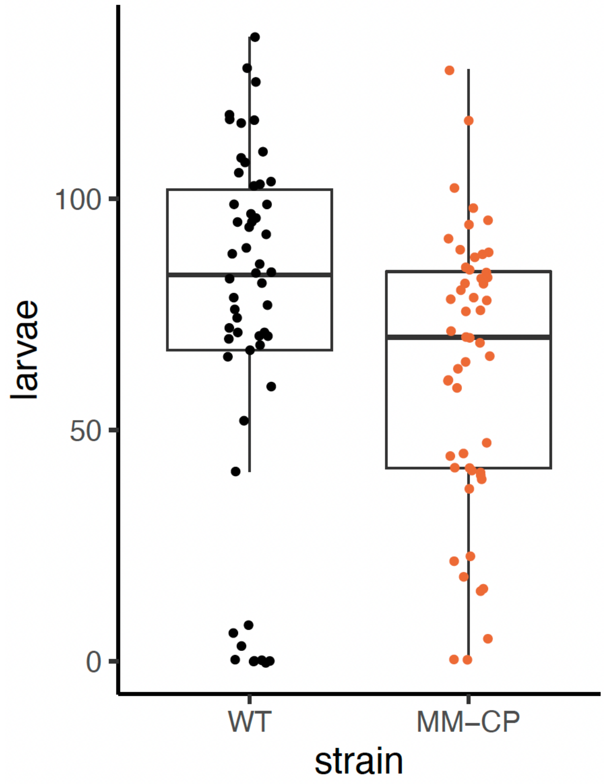

Fig. S4.

Larval output of individual homozygous MM-CP females compared to the wildtype (WT) obtained during the first gonotrophic cycle shows a 15.6% reduction (p=0.079). Data from 3 pooled biological replicates are shown. Statistical significance was determined by a t-test assuming unequal variance.

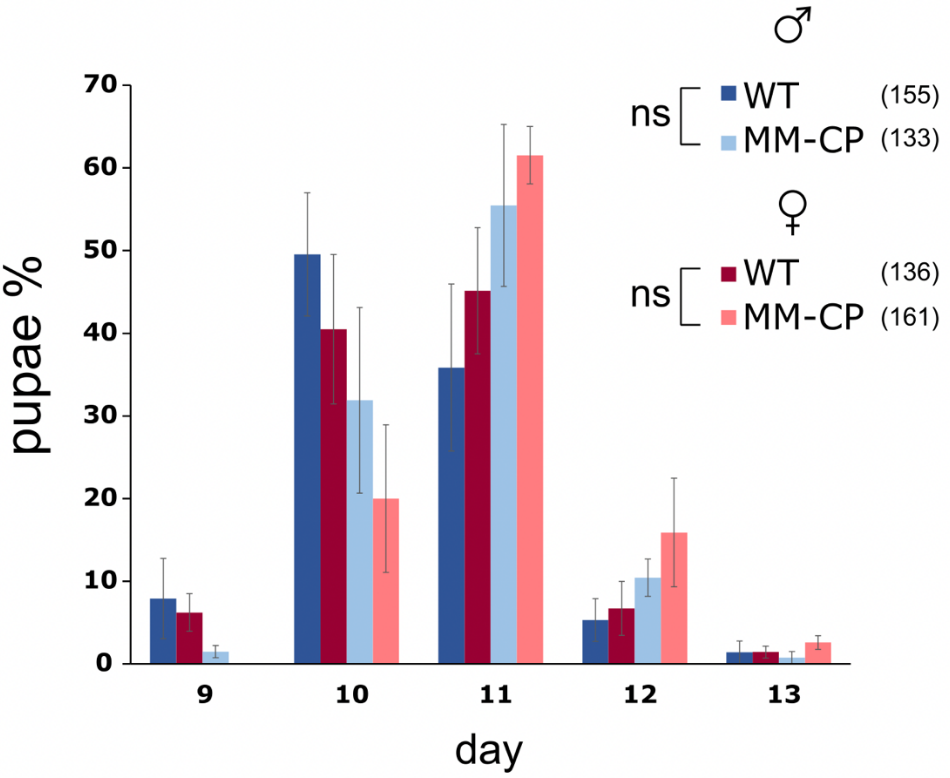

Fig. S5.

Analysis of the time of pupation of MM-CP and wild-type mosquitoes as the percentage of pupae emerging each day. Statistical analysis of average pupation times in males and females was calculated using the Mann-Whitney test.

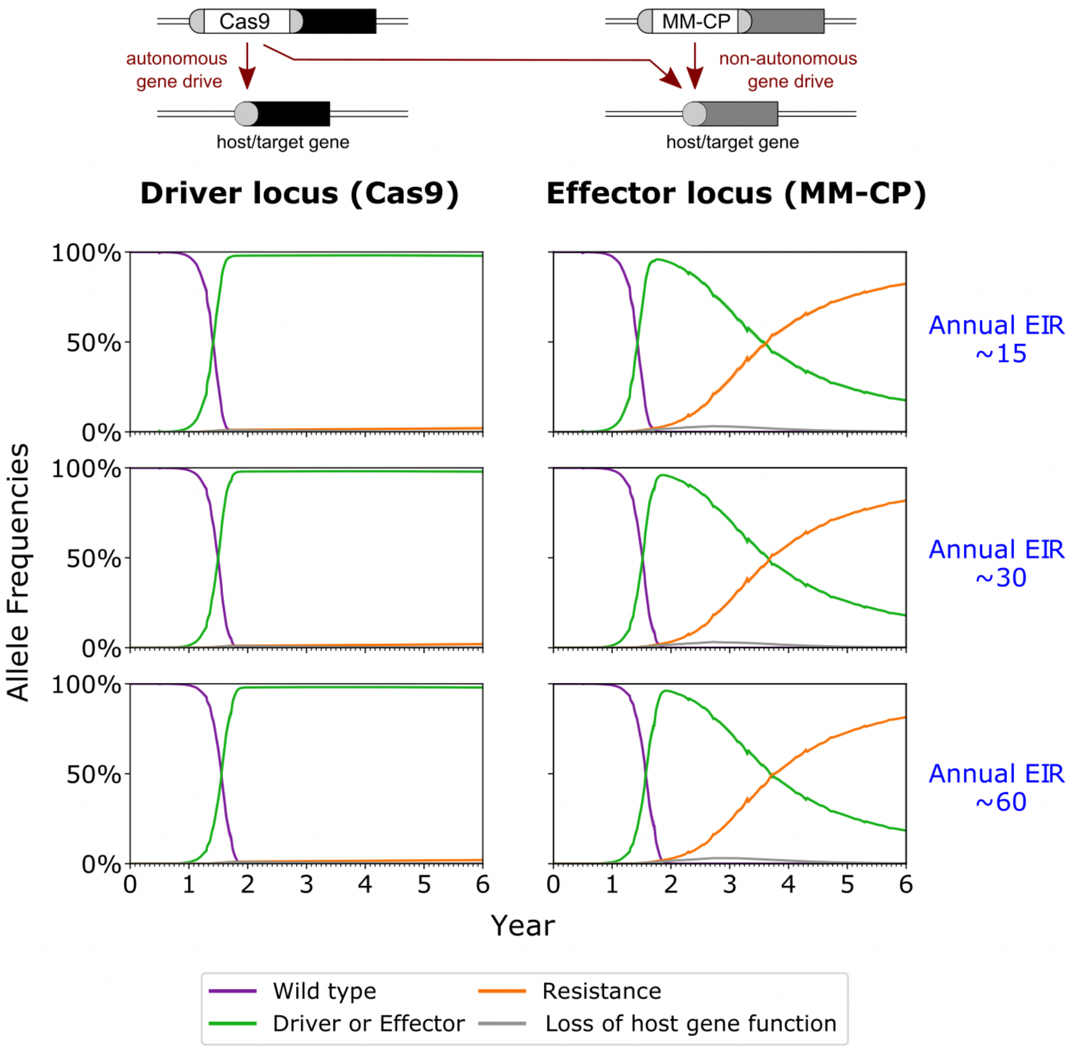

Fig. S6.

The mean time course of allele frequencies after 1000 gene drives mosquitoes homozygous for the driver and effector locus are released 6 months into a 6-year simulation. Each row represents a different transmission intensity. The left column depicts allele frequency at the driver locus and the right column represents allele frequencies at the effector locus. Shown is the mean allele frequency as calculated from 25 stochastic realizations of each scenario.

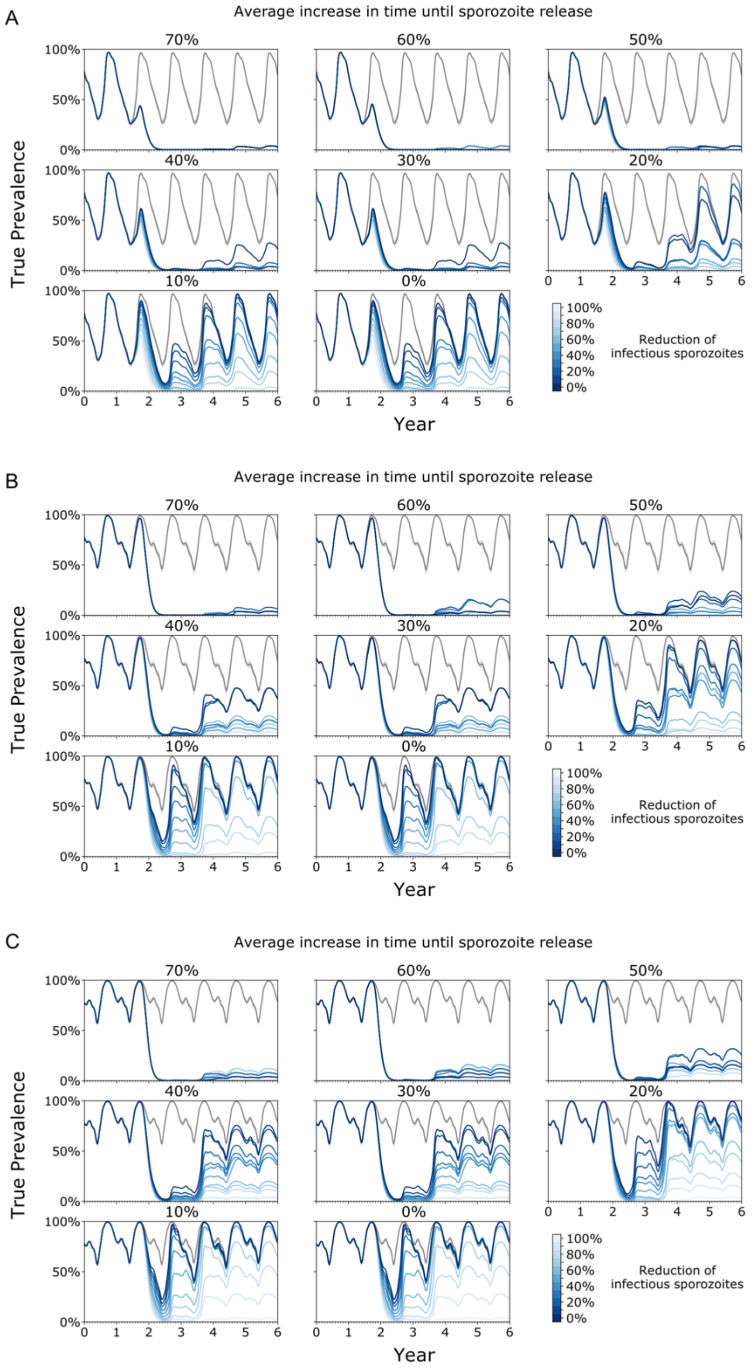

Fig. S7.

The mean time course of true prevalence after 1000 gene drives mosquitoes homozygous for the driver and effector locus are released 6 months into a 6-year simulation. Annual EIR of around 15 (A), 30 (B) and 60 (C) infectious bites per person in an unmitigated scenario. Each panel represents the average increase in time until sporozoites are released while the blue shaded traces each represent a different average reduction in infectious sporozoites. The grey trace represents the unmitigated baseline scenario. There is a temporary reduction in vector numbers when the effector reaches a high frequency because of the fitness costs associated with its expression. This, in conjunction with a reduced lifespan of homozygous females, leads to a temporary reduction in transmission, even when we assume no reduction in infectious sporozoites and no delay in release of sporozoites. This corrects as soon as resistant alleles start to dominate.

			gene counts			*p value*	
**GO.ID**	**Term**	**Condition**	**Annotated**	**Significant**	**Expected**	**elimKS**	**classicFisher**
GO:0046914	transition metal ion binding	6 hours	95	72	59.41	0.004	0.0031
GO:0046872	metal ion binding	6 hours	201	141	125.71	0.106	0.0086
GO:0015318	inorganic m.e. transmembrane transporter activity	20 hours	52	38	27.52	0.00019	0.0018
GO:0015075	ion transmembrane transporter activity	20 hours	59	41	31.23	0.00046	0.0057
GO:0098800	inner mitochondrial membrane protein complex	20 hours	56	39	29.68	0.0021	0.0066
GO:0031966	mitochondrial membrane	20 hours	78	52	41.34	0.4586	0.0073
GO:0005740	mitochondrial envelope	20 hours	80	53	42.4	0.4701	0.0082
GO:0044455	mitochondrial membrane part	20 hours	65	44	34.45	0.384	0.009
GO:0005743	mitochondrial inner membrane	20 hours	72	48	38.16	0.435	0.0099

Table S1.

Enriched GO terms

Fitness & phenotypic parameters				
**Locus**	**Allele combination**	**Daily mortality (%)**	**Fecundity (%)**	**Applied to sex**
driver	WT , D		-2.5	M,F
driver	WT , LGF		-10	M,F
driver	D , D		-5	M,F
driver	D , R		-2.5	M,F
driver	D , LGF		-12.5	M,F
driver	R , LGF		-10	M,F
driver	LGF , LGF		-100	M,F
effector	WT , LGF	+10		M,F
effector	E , E	+30	-14	F
effector	E , LGF	+10		M,F
effector	R , LGF	+10		M,F
effector	LGF , LGF	+100		M,F
Gene drive parameters				
**Locus**	**Allele combination**	**Outcome**	**Probability**	**Allele generated**
driver	D , WT	Gene Drive (autonomous)	0.97	D
		Unmodifed	0.1	WT
		NHEJ to R2	0.1	LGF
		NHEJ to R1	0.1	R
effector	E , WT *(+D)*	Gene Drive (non-autonomous)	0.97	E
		Unmodifed	0.1	WT
		NHEJ to R2	0.1	LGF
		NHEJ to R1	0.1	R

Table S2.
Table of EMOD modelling parameters.

Supplementary file S1. (separate file)
Annotated DNA sequences of transformation vectors pD-MM-CP and pD-Gam1-MM.

Supplementary file S2. (separate file)
DNA oligonucleotide database.
